# A Brain Signaling Framework for Stress-Induced Depression and Ketamine Treatment Elucidated by Phosphoproteomics

**DOI:** 10.3389/fncel.2020.00048

**Published:** 2020-04-07

**Authors:** Yan Xiao, Huoqing Luo, Wen Z. Yang, Yeting Zeng, Yinbo Shen, Xinyan Ni, Zhaomei Shi, Jun Zhong, Ziqi Liang, Xiaoyu Fu, Hongqing Tu, Wenzhi Sun, Wei L. Shen, Ji Hu, Jiajun Yang

**Affiliations:** ^1^Department of Neurology, Shanghai Jiao Tong University Affiliated Sixth People’s Hospital, Shanghai, China; ^2^School of Life Science and Technology, Shanghaitech University, Shanghai, China; ^3^State Key Laboratory of Neuroscience, CAS Center for Excellence in Brain Science and Intelligence Technology, Shanghai Institutes for Biological Sciences, Chinese Academy of Sciences, Institute of Neuroscience, Shanghai, China; ^4^University of Chinese Academy of Sciences, Beijing, China; ^5^Shanghai Institute for Advanced Immunochemical Studies & School of Life Science and Technology, Shanghaitech University, Shanghai, China; ^6^CAS Key Laboratory of Synthetic Chemistry of Natural Substances, Shanghai Institute of Organic Chemistry, Chinese Academy of Sciences, Shanghai, China; ^7^Delta Omics Inc., Baltimore, MD, United States; ^8^Chinese Institute For Brain Research, Beijing, China

**Keywords:** depression, chronic unpredictable mild stress (CUMS), ketamine, LC-MS/MS, phosphoproteomics, brain signaling

## Abstract

Depression is a common affective disorder characterized by significant and persistent low mood. Ketamine, an N-methyl-D-aspartate receptor (NMDAR) antagonist, is reported to have a rapid and durable antidepressant effect, but the mechanisms are unclear. Protein phosphorylation is a post-translational modification that plays a crucial role in cell signaling. Thus, we present a phosphoproteomics approach to investigate the mechanisms underlying stress-induced depression and the rapid antidepressant effect of ketamine in mice. We analyzed the phosphoprotein changes induced by chronic unpredictable mild stress (CUMS) and ketamine treatment in two known mood control centers, the medial prefrontal cortex (mPFC) and the nucleus accumbens (NAc). We initially obtained >8,000 phosphorylation sites. Quantitation revealed 3,988 sites from the mPFC and 3,196 sites from the NAc. Further analysis revealed that changes in synaptic transmission-related signaling are a common feature. Notably, CUMS-induced changes were reversed by ketamine treatment, as shown by the analysis of commonly altered sites. Ketamine also induced specific changes, such as alterations in synapse organization, synaptic transmission, and enzyme binding. Collectively, our findings establish a signaling framework for stress-induced depression and the rapid antidepressant effect of ketamine.

## Introduction

Depression is a devastating disease characterized by a combination of symptoms such as inferiority, loss of appetite, low energy and unexplained discomfort (Gould et al., 2019). This diagnosis occurs at some stage in the life of approximately 16% of the world’s population and is related to severe health and socioeconomic consequences (Li and Vlisides, 2016). The cause of depression has been suggested to be maladaptive changes in specific brain circuits in response to environmental stimuli, such as stressful events. Meanwhile, functional magnetic resonance imaging (fMRI) showed increased activation in response to negative vs. neutral stimuli in the medial prefrontal cortex (mPFC), and increased activation of the nucleus accumbens (NAc) and the amygdala in depression patients (Anand et al., 2005; Harvey et al., 2005; Roberson-Nay et al., 2006). Clinically, the lag time of the therapeutic efficacy is a remarked limit for the conventional antidepressants including selective serotonin reuptake inhibitors (SSRIs) and selective norepinephrine reuptake inhibitors (SNRIs; Dale et al., 2015). Recently, the N-methyl-D-aspartate (NMDA) receptor antagonist ketamine was found to elicit antidepressant effects only several hours after a single administration, and these effects lasted for several days in both major depressive disorder (MDD) patients and animal models of depression (Diazgranados et al., 2010; Autry et al., 2011; Murrough et al., 2013; Zanos et al., 2017; Cui et al., 2018; Yang et al., 2018). Mechanistically, both conventional antidepressants and ketamine promoted neural plasticity, including increased expression of brain-derived neurotrophic factor (BDNF; Moda-Sava et al., 2019), activation of cAMP response element-binding protein (CREB; Duman and Voleti, 2012; Reus et al., 2016), and activation of mammalian target of rapamycin (mTOR) signaling in the mPFC (Li et al., 2010). Rapid and persistent enhancement of neural plasticity, for example, *via* immediate suppression of eukaryotic elongation factor 2 (eEF2) or Ca^2+^/calmodulin-dependent protein kinase II (CaMKII) signaling to induce global protein synthesis in the hippocampus, is also required for the rapid antidepressant activity of ketamine (Monteggia et al., 2013; Taha et al., 2013; Adaikkan et al., 2018). Conversely, impairment of neural plasticity is fundamental for the development of depression (Duman, 2002; Quiroz and Manji, 2002; Liu et al., 2017). However, the cellular basis of depressive disorders is still unclear.

Currently, mass spectrometry (MS)-based proteomics approaches have been used as a powerful tool in systems biology research and have identified many important signaling proteins related to various diseases (Ye et al., 2016; Liu et al., 2018; Wang et al., 2018; Eckert et al., 2019; Noberini and Bonaldi, 2019). Therefore, a comprehensive characterization of phosphorylation events in the whole brain will enable us to obtain an integrated blueprint to understand the cellular mechanism of depression. Herein, we applied this technology along with behavioral and pharmacological investigations to achieve a systemic view of the molecular signaling dysfunctions in depressive conditions and the antidepressant state induced by ketamine. We first demonstrated that the chronic unpredictable mild stress (CUMS) induced depressive-like states, and ketamine produced rapid antidepressant effects in mice, mimicking clinical observations. Then, we identified more than 8,000 phosphorylation sites. Among them, 3,988 sites in the mPFC and 3,196 sites in the NAc were quantifiable. From these experiments, we obtained a systemic overview of the synaptic proteins altered by CUMS in the mPFC and the NAc and found that ketamine reversed these. Altogether, phosphoproteomics combined with pharmacological—approaches helped us to—unveil signaling pathways involved in the phosphorylation-mediated rapid antidepressant effects. Consequently, our approach reveals a novel strategy for the discovery of phosphorylated protein-based therapeutics.

## Materials and Methods

### Animals

All animal care and experimental procedures were approved by the Animal Care and Use Committee of ShanghaiTech University, Shanghai Model Organisms Center, Inc. C57BL/6J mice were housed at 20–22°C and the humidity between 50% and 60%, under a 12:12 h light/dark cycle (light on between 7:00 AM–19:00 PM), Mice had *ad libitum* access to food and water. All behavioral assays were performed on animals 12–16 weeks old which were purchased from Shanghai JieSiJie Experiment Animal Company Limited. Forced Swim Test (FST) and Tail Suspension Test (TST) were performed during the light phase. The Sucrose Preference Test (SPT) began during the dark phase to maximize the consumption of the solution. All behavioral analyses were performed blinded to experimental conditions.

### Drug and Reagents

Ketamine was dissolved in 0.9% saline for intraperitoneal (IP) injection (10 mg/kg). The 0.9% saline was used as the vehicle. EDTA-free protease inhibitor cocktail tablets (#05892791001) and Phosphatase inhibitor cocktail tablets (#04906837001) were purchased from Roche (Basel, Switzerland). Pierce™ BCA Protein Assay Kit (#23225) and anti-phospho-Csnk1a1 (#PA5-36790) were purchased from Thermo Fisher Scientific (Waltham, MA, USA). Titansphere Phos-TiO_2_ kit (#5010-21312) and Monospin C18 (#5010-21701) were purchased from GL Sciences (Japan). Anti-GAPDH (#sc-32233) was purchased from Santa Cruz (Dallas, TX, USA). HRP-conjugated Goat Anti-Rabbit IgG (#D110058) and HRP-conjugated Goat Anti-Mouse IgG (#D110087) were purchased from the Sangon Biotech (Shanghai, China). RNAscope 2.5 HD detection reagent kit (#322350) and Prkcg probe (#417911) were purchased from Advanced Cell Diagnostics (Newark, NJ, USA). Urea (U0631), ammonium bicarbonate (ABC) and other materials were obtained from Sigma–Aldrich (St. Louis, MO, USA) unless otherwise indicated.

### Chronic Unpredictable Mild Stress (CUMS)

The depression model was described previously with a slight modification (Gu et al., 2014). CUMS-induced mice groups were exposed to different stressors for 21 days. The CUMS procedure followed a random weekly schedule of commonly used mild stressors: (1) food or water deprivation (24 h); (2) soiled cages (200 ml water in the sawdust bedding, 24 h); (3) 45° angle cage tilt (24 h); (4) tail clipping (1 h); (5) foot shock (1.75 mA, 40 min); (6) overnight illumination (12 h); (7) social attack (15 min); and (8) restraint in a 50-ml tube (6–8 h). The stressors intensity increased gradually to avoid mice to adapt to stress. After the last CUMS procedure, mice received a series of behavioral tests as indicated. Mice rested at least 2 h between two different behavioral tests.

### Sucrose Preference Test (SPT)

Animals were singly housed and accustomed to two bottles of water for 3 days, followed by two bottles of 2% sucrose for 3 days. Animals were then deprived of water for 24 h and then exposed to two bottles for choice, with one of 1% sucrose and another of water for 2 h in the dark phase. During the test, the positions of the bottle were switched after 1 h to avoid any preference of bottle positions (Opal et al., 2014). Total consumption of each fluid was measured on days 7, 14, 21, 28 during the CUMS, respectively. The sucrose consumption ratio was defined as the total consumption of sucrose divided by the total consumption of water and sucrose. The sucrose preference referred to the average sucrose consumption ratio during the 2 h.

### Forced Swim Test (FST)

Mice were individually placed in a cylinder (20 cm diameter, 40 cm height) filled with water (26–27°C) and swam for six min under normal light (Gigliucci et al., 2013). The water depth was set to 15 cm to prevent the tail or hind limbs of the mouse from touching the bottom of the pool. Mice behaviors were tracked from the top (Shanghai Jiliang Software Technology). The time that mice stay floating or motionless under the actions necessary to maintain balance in the water was defined as “immobile time” and was counted offline by an observer blinded to animal treatment during the last four min in the test.

### Tail Suspension Test (TST)

TST behavior was recorded by a computerized device allowing four animals to be tested at one time. Mice were suspended 50 cm above the floor by adhesive tape placed approximately 1 cm from the tip of the tail in a soundproof chamber. Meantime, the activities of mice were recorded using cameras and the total duration of immobility was analyzed during the last four min in the six min by an observer blinded to animal treatment test.

### Ketamine Experiment

Ketamine was dissolved in saline (0.9%) and administered intraperitoneally (IP). The concentration of ketamine was 10 mg/kg for mice. After ketamine delivery, CUMS mice were respectively used for SPT or FST and TST behavioral assays after 1 or 24 h.

### Phosphoproteomics Sample Preparation

In phosphoproteomics experiments, each experimental group included four mice. The saline and ketamine (10 mg/kg) were injected intraperitoneally (IP) into mice. After 1 h, animals were killed by cervical displacement, and then the brains were removed from the skull and microdissected immediately. The mPFC and NAc were flash-frozen in liquid nitrogen after dissection. Samples were then stored at −80°C for subsequent phosphoproteomic analysis. Frozen brain tissues were transferred into tubes, which contained 200 μl of lysis buffer (8 M urea, 50 mM ABC, pH 8) with protease inhibitor (10 ml/tablet) and phosphatase inhibitor (10 ml/tablet) added. Samples were then sonicated for 2 min on ice. The supernatant was extracted after centrifuged for at 4°C for 5 min at 14,000 *g*. The bicinchoninic acid (BCA) assay was used to determine the protein concentration of the lysates. Next, dithiothreitol (DTT, 5 mM) was added to the same quality samples and put it on a 37°C shaker for 2 h. After cooling to room temperature, iodoacetamide (IAM, 1 M) was added and protected from light for 40 min. Samples were treated with trypsin (#V5113, Promega) at 37°C overnight and the ratio of protein-to-enzyme was 50:1. The protein digestion was stopped by adding 10% trifluoroacetic acid (TFA) until a pH <2 was reached. Then, the supernatant was collected after centrifuged at 25°C for 5 min at 14,000 *g*. The peptides were desalted using Monospin C18 according to the protocol. Briefly, tips were washed with 100 μl of buffer B (0.1% formic acid, 50% acetonitrile) followed by 100 μl of buffer A (0.1% formic acid). Peptides were then loaded onto the tips and centrifuged at 1,000 *g* for 5 min and repeated once. Then, the samples were eluted with buffer B after washed with buffer A. Next, the resulting peptide solution dried with a SpeedVac apparatus. After this step, the phosphopeptides were enriched and then purified using the Titansphere Phos-TiO_2_ kit according to the protocol (Forget et al., 2018). In Brief, tips were washed with 100 μl of TiO_2_ buffer A (0.4% TFA, 80% acetonitrile) followed by 100 μl of TiO_2_ buffer B (25% lactic acid, 75% TiO_2_ buffer A). Peptides were then loaded onto the tips and centrifuged at 1,000 *g* for 5 min and repeated once. The 100 μl of TiO_2_ buffer B and 100 μl of TiO_2_ buffer A were used to wash the tips, respectively. Then, the samples were eluted by the elution buffer (5% NH_4_OH, 40% acetonitrile). Finally, the eluates were dried by a SpeedVac apparatus and then analyzed by LC-MS/MS.

### LC-MS/MS Analysis of Enriched Peptides

Peptides were resuspended with 5 μl mobile phase A (0.1% formic acid in 2% acetonitrile), then separated by nanoLC (Easy-nLC 1000 system) and analyzed by on-line electrospray tandem mass spectrometry (Q Exactive HF-X, Thermo Fisher Scientific). Three microliter peptide sample was loaded and separated by the analytical column (75 μm × 50 cm, Thermo Fisher Scientific) using a flow rate of 250 nl/min. A 90-min gradient was set up using mobile phase A and mobile phase B (0.1% formic acid in 98% acetonitrile), among them, from 4% to 28% B in 79-min, 28% to 60% B in 1-min, and climbing to 90% B in 1-min, then holding at 90% for the last 9-min. The electrospray was used to ionize the peptides at 2.1 kV. A completed MS spectrum in the range of 375–1,500 m/z was obtained at a resolution of 120,000 at m/z 200 and a maximum ion accumulation time of 20 ms. 30 s was the dynamic exclusion setting parameter and the 15,000 was the resolution for HCD MS/MS spectra at m/z 200. The AGC of MS1 was set at 3E6 and that of MS2 was 1E5. HCD selected the 20 strongest ions above the 2.5E4 count threshold for the fragmentation, and the maximum ion accumulation time was 80 ms. MS2 isolation width was 1.6 m/z units. MS/MS removed single and unassigned charged ions. The normalized collision energy was 27% for HCD.

### MS Data Analysis

Tandem mass spectra were processed by MaxQuant (v.1.6.5.0), and the database used for database searches was the mouse protein database (release 2016_07, 49,863 sequences). There allowed two missed cleavage sites for the enzyme of trypsin. The mass error of the precursor ions was 5 ppm and that of the fragment ions was 0.02 Da. The fixed modification was carbamidomethylation on Cys, and the variable modifications were oxidation (M), acetylation (Protein N-term), and phosphorylation (STY). The thresholds of false discovery rate (FDR) for protein, peptide, and modification sites were set at 1% and 7 was the minimum length of the peptide. The three options including “Second peptides,” “Match between runs” and “Dependent peptides” selected the enable and all other parameters retained the setting of default values. Moreover, Student’s *t*-test was performed to test the significance of the differences between each comparison group.

### Bioinformatics Analysis

We only chose the commonly identified phosphorylation sites across samples for further quantitative analysis for each type of brain parts. Then, the two comparisons have been carried out: CUMS + saline vs. Control + saline, CUMS + ketamine vs. CUMS + saline, and CUMS + ketamine vs. Control + saline. We chose the ratio of 1.2-fold change and the probability of 0.05 as a cut-off for differentially expressed proteins or sites as indicated. The multi-omics data analysis tool, OmicsBean, was used to analyze the obtained phosphoproteins data^1^, in which distributions in biological process, cell component and molecular function were assigned to each protein based on Gene Ontology (GO) categories. Protein-protein interaction (PPI) analysis was performed also using OmicsBean. The DAVID (Database for Annotation, Visualization, and Integrated Discovery) functional annotation tool was used to analyze the Kyoto Encyclopedia of Genes and Genomes (KEGG; Kanehisa et al., 2014) pathway^2^ mapping of phosphoproteins (Huang da et al., 2009).

### Western Blot Analysis

For western blot analysis, the protein samples were resolved and transferred onto nitrocellulose filter (NC) membranes. After blocking with 5% BSA in TBS-tween 20 at room temperature for 2 h, the membranes were performed using specific primary antibody anti-phospho-Csnk1a1 (1:2,000) and anti-GAPDH (1:1,000) at 4°C overnight. The membranes were then incubated with HRP-conjugated Goat Anti- Rabbit IgG (1:2,000) and HRP-conjugated Goat Anti-Mouse (1:2,000) at room temperature for 2 h. Western blot signals were quantified using Amersham Imager 600 (GE Healthcare), and band signals were expressed as relative protein amounts compared to GAPDH.

### *In situ* Hybridization

The RNAscope 2.5 HD Detection Kit was used to RNAscope *in situ* hybridization according to the manufacturer’s protocol. After the mice were anesthetized, they were perfused with PBS followed by 4% PFA. The brains of the mice were removed and immediately placed in freshly prepared 4% PFA, and fixed at 4°C for 24 h. Brain tissues were immersed in 1× PBS containing 30% sucrose for 2 days at 4°C until the tissues sink to the bottom of the container. Then the brain tissues embedded the OCT and placed at -20°C for 1 h. Sections of 14 μm thickness were prepared using a cryostat. The slides were washed in 1× PBS for 5 min to remove OCT. Two to four drops of hydrogen peroxide were added to each section and incubated at room temperature for 10 min. The slides were immersed in boiling 1× target repair reagent to repair 5 min and then washed with fresh distilled water twice followed by 100% ethanol. Two to four drops of protease plus were added in the hydrophobic circle on each brain slice and incubated at 40°C for 30 min. The slides were washed with fresh distilled water twice. And then about four drops of Prkcg probe were added to each brain slice in the hydrophobic circle and incubated at 40°C for 2 h. The slides were washed with 1× wash buffer 2 times at room temperature for 2 min. Amp 1 (incubated at 40°C for 30 min), Amp 2 (incubated at 40°C for 15 min), Amp 3 (incubated at 40°C for 30 min), Amp 4 (incubated at 40°C for 15 min), Amp 5 (incubated at room temperature for 30 min), and Amp 6 (incubated at room temperature for 15 min) were performed in sequence, and finally the RED working solution was added to detect the signal.

### Statistical Analysis

Statistical analysis was carried out using the GraphPad Prism 8.0 software. No methods of randomization were used to allocate animals to experimental groups. All experiments were blind to the experimenter. Two-tailed Student’s *t*-test (95% confidence), one-way ANOVA and two-way ANOVA, with Bonferroni’s *post hoc* multiple comparison test, were performed as required, respectively. Two by two comparisons were two-tailed. *P-value < 0.05* was considered statistically significant.

## Results

### Experimental Workflow and Depression-Like States Induced by CUMS

**Figure 1:**
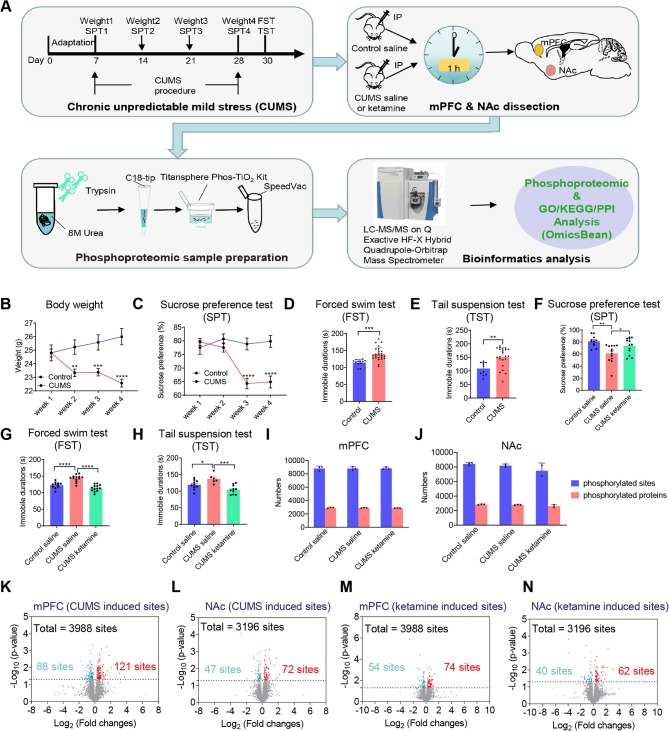
Overview of the experimental workflow, related behavioral tests, and summary of phosphorylation changes induced by chronic unpredictable mild stress (CUMS) and ketamine. **(A)** The protocol of using CUMS to induce depression-like state and performing phosphoproteomic analysis afterward. First, the mice were subjected to CUMS protocol as indicated. SPT, sucrose preference test; FST, forced swim test; TST, tail suspension test. Second, ketamine or saline was administrated intraperitoneally (IP) to CUMS and control mice as indicated. All mice were killed 1-h after injection (*n* = 4 per group). Two brain regions (mPFC, NAc) were dissected and processed for phosphoproteomic analysis. mPFC, medial prefrontal cortex; NAc, nucleus accumbens. Third, proteins for phosphoproteomic analysis were tryptic digested in solution, and peptides were loaded onto the tips using Monospin C18 and phosphopeptides from each fraction were enriched by Titansphere Phos-TiO_2_ Kit. Finally, the samples were subjected to LC and Q-Exactive HF-X analysis, following by bioinformatics analysis [Gene Ontology (GO)/Kyoto Encyclopedia of Genes and Genomes (KEGG)/protein-protein interaction (PPI) analysis]. **(B)** Bodyweight changes during the CUMS protocol (Control, *n* = 12; CUMS, *n* = 25, two-way ANOVA, **P < 0.01, ***P < 0.001, ****P < 0.0001 compared with control group). **(C–E)** Behavioral evaluations after the CUMS to confirm the depression-like state. The preference value in the SPT was shown in (**C**; Control, *n* = 12; CUMS, *n* = 25, two-way ANOVA, ****P < 0.0001 compared with control group). The duration of immobility in the FST was shown in (**D**; Control, *n* = 12; CUMS, *n* = 25, *t*-test, ***P < 0.001 compared with control group). The duration of immobility in the TST was shown in (**E**; Control, *n* = 11; CUMS, *n* = 22, *t*-test, **P < 0.01 compared with control group). **(F–H)** Behavioral evaluations after ketamine injection (IP) to CUMS mice to confirm the antidepressant effect. The preference value in the SPT was shown in (**F**; Control + saline, *n* = 11; CUMS + saline, *n* = 13; CUMS + ketamine, *n* = 13, one-way ANOVA, **P < 0.01 for CUMS + saline compared with Control + saline; *P < 0.05 for CUMS + saline compared with CUMS + ketamine). The duration of immobility in the FST was shown in (**G**; Control + saline, *n* = 11; CUMS + saline, *n* = 13; CUMS + ketamine, *n* = 13, one-way ANOVA, ****P < 0.0001 for CUMS + saline compared with Control + saline; ****P < 0.0001 for CUMS + saline compared with CUMS + ketamine). The duration of immobility in the TST was shown in (**H**; Control + saline, *n* = 10; CUMS + saline, *n* = 6; CUMS + ketamine, *n* = 10, one-way ANOVA, *P < 0.05 for CUMS + saline compared with Control + saline; ***P < 0.001 for CUMS + saline compared with CUMS + ketamine). All data are shown as mean ± SEM. **(I,J)** The number of identified phosphorylated sites and proteins in different groups (Control + saline, CUMS + saline, and CUMS + ketamine) in the mPFC **(I)** and the NAc **(J)**, respectively. **(K,L)** The number of quantified differential phosphorylated sites between CUMS and controls without treatment in the mPFC **(K)** and the NAc **(L)**. **(M,N)** The number of quantified differential phosphorylated sites from CUMS mice between ketamine and saline injections in the mPFC **(M)** and the NAc **(N)**. The ratio of 1.2-fold change and the probability of 0.05 as a cut-off for differential expressed sites with statistical significance. The differential phosphorylated sites are shown in red (up-regulated) and blue (down-regulated).

To investigate the phosphorylation changes related to depression and the antidepressant effect of ketamine, we used CUMS to induce depression-like states in mice (Deng et al., 2015; Jin et al., 2015; Ma et al., 2016) and then treated with ketamine before performing the phosphoproteomics analysis (Figure 1A). Briefly, the mice were subjected to unpredictable mild stress, including food or water deprivation, soiled cages, 45° angle cage tilt, tail clipping, foot shock, overnight illumination, social attack, and restraint in a 50-ml tube for 21 days. Age-matched wild-type littermates that did not receive the stress treatment were used as controls. The body weight and sucrose preference were monitored weekly during the protocol (Figure 1A). As expected, the CUMS group showed progressive decreases in body weight and sucrose preference (Figures 1B,C), which are the typical signs of depression (Burstein and Doron, 2018). Furthermore, we performed the forced swim test (FST) and tail suspension test (TST) to assess the depressive state. As expected, the CUMS group exhibited an increased immobility time during the FST (Figure 1D) and TST (Figure 1E), indicative of a depression-like state (Castagné et al., 2011). After successfully establishing the CUMS model, we intraperitoneally (IP) administered saline to the control group and saline or ketamine to the CUMS group (Figure 1A). Notably, 1 h after ketamine injection, the sucrose preference was significantly reversed to control levels (Figure 1F). The immobilization phenotypes in the FST and TST were also corrected 24-h post-injection (Figures 1G,H; Franceschelli et al., 2015).

Since we have confirmed the depression-like behaviors and the antidepressant effect of ketamine, 1-h after ketamine or saline injection, we quickly dissected two brain areas, the mPFC and the NAc, which have been associated with mood control (Rive et al., 2013; Floresco, 2015; Lieberman et al., 2019). The brain tissues from the two areas in a group of four mice were then homogenized and digested with trypsin. The phosphopeptides were extracted according to the procedure shown in Figure 1A. To ensure the reliability of the quantitative profiling results, the samples were prepared and the fractions were collected from three biological replicates per group. All samples were subjected to parallel label-free phosphoproteomics analysis using liquid chromatography-tandem mass spectrometry (LC-MS/MS). Unfortunately, one of the biological triplicates from the NAc group treated with ketamine was discarded due to poor sample processing.

### Systematic Examination of Phosphorylation Changes Induced by CUMS and Ketamine

After phosphoproteomics analysis, we successfully identified ~8,800 different phosphorylated sites mapping to ~2,900 proteins from the mPFC groups (Figure 1I) and ~8,000 phosphorylated sites mapping to ~2,750 proteins from the NAc groups (Figure 1J). To test the reproducibility of our study, we performed Pearson correlation analysis between each biological replicate and discovered that the Pearson correlation between the experimental repeats was >0.85 in the mPFC (Supplementary Figure S1A) and >0.79 in the NAc (Supplementary Figure S1B), indicating robust reproducibility. In the meantime, we analyzed the overlapped phosphorylation sites between each replicate within a group and found that the percentage of the overlapped phosphorylation sites identified between every two repeats was approximately 80% (Supplementary Table S1). Next, we defined the differential phosphorylation sites between the CUMS group and the control as CUMS-induced phosphorylation and those between the CUMS group treated with ketamine and saline as ketamine-induced phosphorylation. We found that 3,988 and 3,196 of the identified phosphorylation sites were quantifiable in the mPFC and the NAc, respectively. Then, the ratio of 1.2-fold change and the probability of 0.05 as a cut-off for differential expressed sites with statistical significance. Therefore, for CUMS-induced changes, we quantified 209 differential phosphorylation sites from the mPFC and 119 differential phosphorylation sites from the NAc. There were 121 up-regulated and 88 down-regulated phosphorylation sites from the mPFC (Figure 1K; Supplementary Data Sheet S1), and 72 up-regulated and 47 down-regulated sites from the NAc (Figure 1L; Supplementary Data Sheet S1). For ketamine-induced changes, we quantified 128 differential phosphorylation sites from the mPFC, of which 74 were up-regulated and 54 were down-regulated (Figure 1M; Supplementary Data Sheet S1), and we quantified 102 differential phosphorylation sites from the NAc, of which 62 were up-regulated and 40 were down-regulated (Figure 1N; Supplementary Data Sheet S1). Moreover, we analyzed the differential phosphorylation between CUMS + ketamine vs. Control + saline. We quantified 270 differential phosphorylation sites from the mPFC, of which 173 were up-regulated and 97 were down-regulated (Supplementary Figure S2A; Supplementary Data Sheet S1), and quantified 108 differential phosphorylation sites from the NAc, of which 69 were upregulated and 39 were down-regulated (Supplementary Figure S2B; Supplementary Data Sheet S1).

### Protein-Protein Interaction (PPI) Networks for CUMS- and Ketamine-Induced Changes

**Figure 2:**
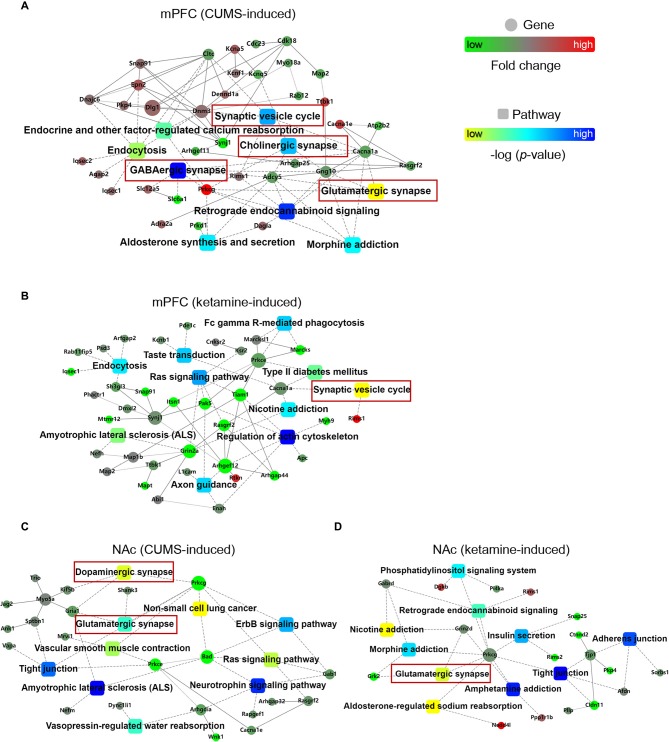
PPI networks for CUMS- and ketamine-induced changes. **(A,B)** The PPI networks for proteins corresponding to quantified phosphorylation sites in the mPFC induced by CUMS **(A)** and ketamine **(B)**. **(C,D)** The PPI networks for proteins corresponding to quantified phosphorylation sites in the NAc induced by CUMS **(C)** and ketamine **(D)**. The PPI analysis is based on fold changes of genes expression, PPIs, and KEGG pathway enrichments. Fold changes of genes are indicated by circle nodes with gradient color (red, up-regulated; green, down-regulated). Rectangles indicate KEGG pathways, which are colored with a yellow to the blue gradient (smaller to larger *p*-values, respectively). The scales are indicated next to PPI networks. The red boxes represent the pathways associated with different synapses.

After the quantification of differential phosphorylation, we wonder whether there are interactions between the proteins carrying these quantified sites as it might help to identify key signaling pathways for depression induction and its treatment. Therefore, we used the OmicsBean analysis tool to investigate PPI networks. Interestingly, we found several pathway hubs in the PPI networks. For example, there were nine pathways induced by CUMS in the mPFC: “Synaptic vesicle cycle,” “Endocrine and other factor-regulated calcium reabsorption,” “Endocytosis,” “Cholinergic synapse,” “GABAergic synapse,” “Glutamatergic synapse,” “Retrograde endocannabinoid signaling,” “Aldosterone synthesis and secretion,” and “Morphine addiction” (Figure 2A). A total of 10 pathways were induced by ketamine in the mPFC: “Fc gamma R-mediated phagocytosis,” “Taste transduction,” “Endocytosis,” “Ras signaling pathway,” “Type II diabetes mellitus,” “Synaptic vesicle cycle,” “Nicotine addiction,” “Amyotrophic lateral sclerosis (ALS),” “Regulation of actin cytoskeleton,” and “Axon guidance” (Figure 2B). In the NAc, there were also 10 pathways induced by CUMS: “Dopaminergic synapse,” “Non-small cell lung cancer,” “Glutamatergic synapse,” “ErbB signaling pathway,” “Vascular smooth muscle contraction,” “Tight junction,” “Ras signaling pathway,” “ALS,” “Neurotrophin signaling pathway,” and “Vasopressin-regulated water reabsorption” (Figure 2C). A total of 10 pathways were induced by ketamine: “Phosphatidylinositol signaling system,” “Retrograde endocannabinoid signaling,” “Nicotine addiction,” “Insulin secretion,” “Morphine addiction,” “Adherens junction,” “Glutamatergic synapse,” “Tight junction,” “Amphetamine addiction,” and “Aldosterone-regulated sodium reabsorption” (Figure 2D).

Furthermore, we wonder whether there is a common feature in these hubs across brain regions and treatments. Interestingly, we found “synapse” as a shared hub feature. As follow, the CUMS-induced PPI hubs in the mPFC included “Glutamatergic synapse,” “GABAergic synapse,” “Cholinergic synapse,” and “Synaptic vesicle cycle” (Figure 2A). The ketamine-induced PPI hub in the mPFC included the “Synaptic vesicle cycle” (Figure 2B). Similarly, the CUMS-induced PPI hubs in the NAc included “Glutamatergic synapse” and “Dopaminergic synapse” (Figure 2C). The ketamine-induced PPI hub in the NAc included “Glutamatergic synapse” (Figure 2D). Thus, our data suggest that synaptic function changes might be an important hallmark for depression progression and treatment, which is consistent with previous reports (Elizalde et al., 2010; Li et al., 2011; Duman and Aghajanian, 2012; Duman et al., 2016).

### Synaptic Signaling Changes Induced by CUMS and Ketamine

**Figure 3:**
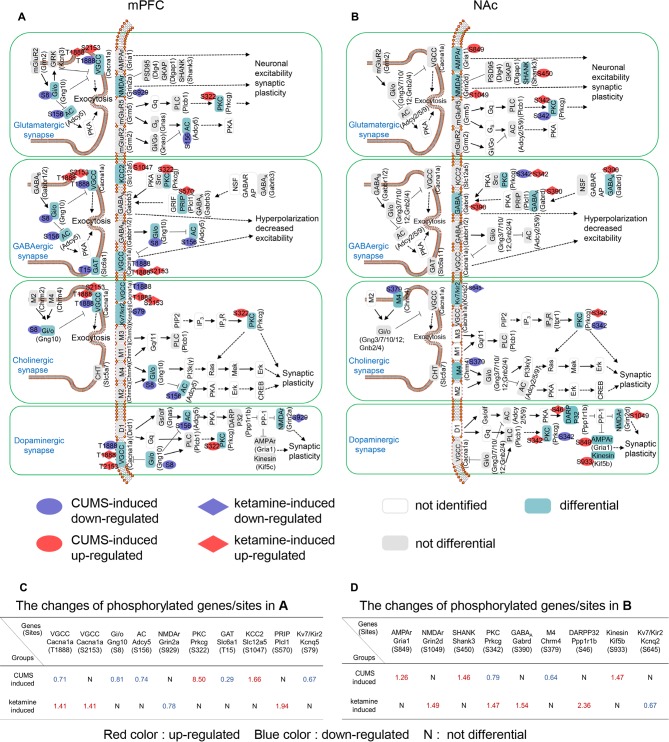
Summary of CUMS- and ketamine-induced signaling pathways at synapses. **(A,B)** Proteins assigned to different KEGG pathways, with particular phosphorylated sites of each protein indicated in ovals (CUMS-induced) and rhombuses (ketamine-induced). The corresponding genes were shown in brackets. The aqua green, gray, and white rectangles indicate differential, not differential, and not identified phosphoproteins in our study, respectively. Four types of synapses were analyzed, including the glutamatergic synapse, the GABAergic synapse, the cholinergic synapse, and the dopaminergic synapse. Red and blue ovals indicate up-regulated and down-regulated phosphorylation induced by CUMS in the mPFC, respectively. Red and blue rhombuses demonstrate up-regulated and down-regulated phosphorylation induced by CUMS and ketamine in the NAc, respectively. **(C)** The phosphorylation list for **(A)**, where red, blue colors and N indicate up-, down-regulated, and not differential, respectively. **(D)** The phosphorylation list for **(B)**, where red, blue colors and N indicate up-, down-regulated, and not differential, respectively.

To unveil the critical synapse-related signaling pathways identified from the PPIs, we classified the relevant protein phosphorylation sites according to their synaptic types, which included glutamatergic, GABAergic, cholinergic and dopaminergic (Figures 3A,B). For glutamatergic synapses, Cacna1a (T1888), Gng10 (S8), and Adcy5 (S156) were down-regulated by CUMS, while Prkcg (S322) was up-regulated in the mPFC (Figure 3A). Grin2a (S929) was down-regulated by ketamine, while Cacna1a (T1888; S2153) was up-regulated in the mPFC (Figure 3A). Gria1 (S849) and Shank3 (S450) were up-regulated, while Prkcg (S342) was down-regulated by CUMS in the NAc (Figure 3B). Grin2d (S1049) and Prkcg (S342) were up-regulated by ketamine in the NAc (Figure 3B). These genes are involved in regulating synaptic plasticity and postsynaptic excitability (Lee et al., 2000; Shuvaev et al., 2011; Ryan et al., 2013; Perszyk et al., 2016; Kellermayer et al., 2018). For GABAergic synapses, Cacna1a (T1888), Gng10 (S8), Adcy5 (S156), and Slc6a1 (T15) were down-regulated, while Prkcg (S322) and Slc12a5 (S1047) were up-regulated by CUMS in the mPFC (Figure 3A). Cacna1a (T1888; S2153) and Plcl1 (S570) were up-regulated by ketamine in the mPFC (Figure 3A). Prkcg (S342) was down-regulated by CUMS in the NAc (Figure 3B). Gabrd (S390) and Prkcg (S342) were up-regulated by ketamine in the NAc (Figure 3B). These genes are involved in regulating postsynaptic hyperpolarization (Yanagihori et al., 2006; Oláh et al., 2009; Shuvaev et al., 2011; Schwale et al., 2016). For cholinergic synapses, Cacna1a (T1888), Gng10 (S8), Adcy5 (S156), and Kcnq5 (S79) were down-regulated, while CUMS up-regulated Prkcg (S322) in the mPFC (Figure 3A). Cacna1a (T1888; S2153) was up-regulated by ketamine in the mPFC (Figure 3A). Chrm4 (S379) and Prkcg (S342) were down-regulated by CUMS in the NAc (Figure 3B). Prkcg (S342) was up-regulated, while Kcnq2 (S645) was down-regulated by ketamine in the NAc (Figure 3B). These genes are involved in regulating synaptic plasticity (Shuvaev et al., 2011; Niday et al., 2017). For dopaminergic synapses, Cacna1a (T1888), Gng10 (S8), and Adcy5 (S156) were down-regulated, while CUMS up-regulated Prkcg (S322) in the mPFC (Figure 3A). Grin2a (S929) was down-regulated, while Cacna1a (T1888; S2153) was up-regulated by ketamine in the mPFC (Figure 3A). Prkcg (S342) was down-regulated, while CUMS up-regulated Gria1 (S849) and Kif5b (S933) in the NAc (Figure 3B). Prkcg (S342), Grin2d (S1049) and Ppp1r1b (S46) were up-regulated by ketamine in the NAc (Figure 3B). These genes are involved in regulating synaptic plasticity (Calabresi et al., 2000; Lee et al., 2000; Shuvaev et al., 2011; Ryan et al., 2013; Perszyk et al., 2016; Kellermayer et al., 2018).

Intriguingly, protein kinase C gamma (Prkcg), which has been implicated in long-term alterations in synaptic efficacy (Saito and Shirai, 2002; McNamara and Lenox, 2004), was drastically enriched by CUMS induction in the mPFC (8.5-fold, Figure 3C) and by ketamine in the NAc (1.47-fold, Figure 3D) and is involved in all four synaptic types (Figures 3A,B). Another highly enriched phosphoprotein was protein phosphatase 1 regulatory subunit 1B (Ppp1r1b), also known as dopamine- and cAMP-regulated neuronal phosphoprotein (DARPP-32), which was enriched 2.4-fold in the NAc by ketamine treatment (Figure 3D). Interestingly, Ppp1r1b has been shown to play an important role in long-term synaptic plasticity in the NAc (Calabresi et al., 2000; Stipanovich et al., 2008). Overall, our data identified the critical changes in synaptic signaling induced by CUMS and ketamine.

### Bioinformatics Analysis of the Shared and Differential Phosphorylation Changes Between the CUMS-Induced and Ketamine-Induced Groups

**Figure 4:**
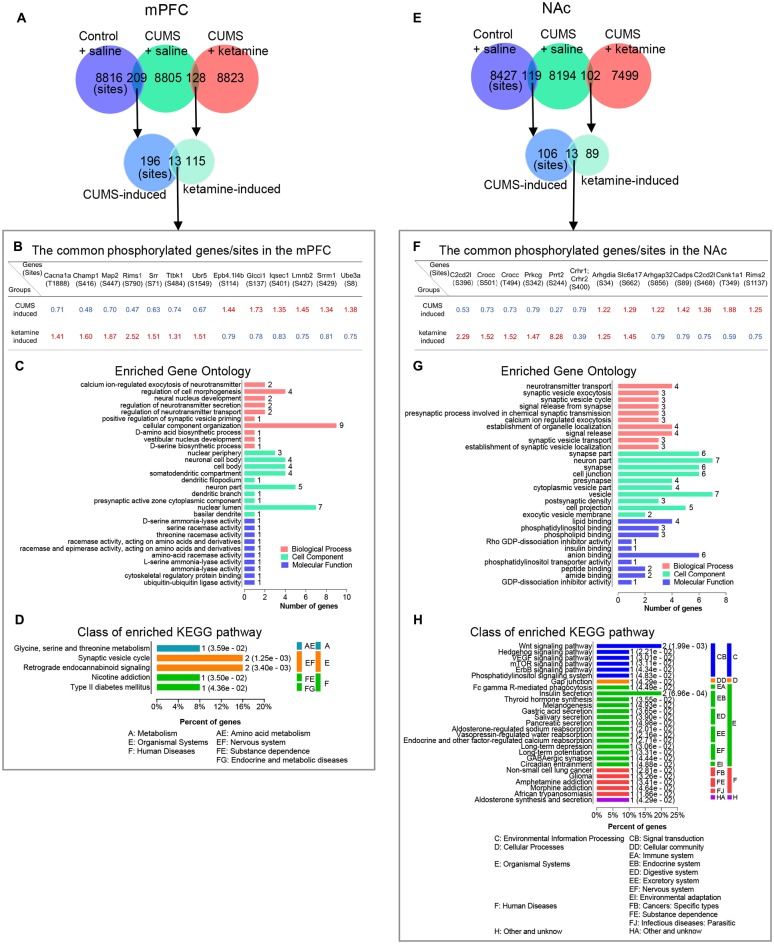
Bioinformatics analysis of the overlapped phosphorylation changes between CUMS- and ketamine-induced. **(A)** The overlapping of identified phosphorylated sites for indicated experimental groups in the mPFC. **(B–D)** Bioinformatics analysis of the proteins corresponding to the common phosphorylation sites between CUMS-induced and ketamine-induced in the mPFC. The genes/sites list, GO analysis, and KEGG pathway analysis are shown in **(B–D)**, respectively. **(E)** The overlapping of identified phosphorylated sites for indicated experimental groups in the NAc. **(F–H)** Bioinformatics analysis of the proteins corresponding to the common phosphorylation sites between CUMS-induced and ketamine-induced in the NAc. The genes/site lists, GO analysis, and KEGG pathway analysis are shown in **(F–H)**, respectively. The red and blue colors indicate up- and down-regulated in **(B,F)**, respectively. Enriched GO analysis shows the 10 most significantly enriched terms of biological process, cell component, and molecular function, respectively. Enriched KEGG pathway analysis shows the number of involved genes in a specific pathway, and corresponding *p*-values are shown on the right side of the column.

As the shared and differential phosphorylation changes induced by CUMS and ketamine might be important for the antidepressant effect of ketamine, we analyzed these changes by (GO: biological process, cell component, and molecular function) and KEGG pathways using the OmicsBean analysis tool. First, we found that 13 phosphorylation sites overlapped between the CUMS-induced and ketamine-induced changes in the mPFC (Figures 4A,B). Interestingly, opposite changes for these overlapping sites were observed between the two groups (Figure 4B). Seven sites down-regulated by CUMS and up-regulated by ketamine, while the remaining six sites were changed in the opposite direction (Figure 4B), suggesting that the altered phosphorylation induced by CUMS was corrected by ketamine treatment. Among them, regulating synaptic membrane exocytosis protein 1 (Rims1) is a member of the Ras superfamily of genes and plays an important role in neurotransmitter release (Wang et al., 1997; Calakos et al., 2004). Rims1 had the largest fold change, with approximately half-fold repression by CUMS and a 2.5-fold enrichment by ketamine (Figure 4B). Moreover, microtubule-associated protein 2 (Map2), a protein that stabilizes microtubule activity and regulates neuronal axons and dendritic microtubule networks (Sánchez et al., 2000), was also repressed by CUMS and enriched by ketamine (Figure 4B). Serine racemase (Srr), a D-serine synthesis enzyme involved in the regulation of synaptic plasticity (Miya et al., 2008), showed similar changes to Map2 (Figure 4B). Next, we performed GO analysis and found that the top enriched categories were “cellular component organization,” followed by “neuron part” and “nuclear lumen” (Figure 4C). Besides, we performed KEGG pathway analysis and found that “Synaptic vesicle cycle” and “Retrograde endocannabinoid signaling” were the top enriched categories (Figure 4D). All other categories from the GO and KEGG analyses are shown in (Figures 4C,D). Additionally, we analyzed CUMS induced sites with phosphorylation levels precisely restored to control levels by ketamine treatment. We obtained 10 phosphorylation sites in mPFC (Supplementary Table S2) and eight sites in the NAc (Supplementary Table S3).

Similarly, we found 13 phosphorylation sites that overlapped between CUMS-induced and ketamine-induced changes in the NAc (Figures 4E,F). Among them, 10 sites were regulated in the opposite direction by CUMS and ketamine (Figure 4F), with five sites downregulated and five sites upregulated by CUMS. The opposite changes were induced by ketamine (Figure 4F). These results confirm that the altered phosphorylation by CUMS was reversed by ketamine treatment. Interestingly, several synaptic plasticity genes, including Proline-rich transmembrane protein 2 [Prrt2 (Michetti et al., 2017)] and Calcium-dependent secretion activator [Cadps (Shinoda et al., 2016)], were involved. Further bioinformatics analysis showed that the top enriched GO annotations were “neuron part” and “vesicle,” followed by “synapse part,” “synapse,” “cell junction,” and “anion binding” (Figure 4G), suggesting that synaptic changes might be important for the depression-like state and the antidepressant effect of ketamine. From the KEGG pathway analysis, the “Wnt signaling pathway” and “Insulin secretion” were the top enriched categories (Figure 4H). All other categories from the GO and KEGG analyses are shown in Figures 4G,H.

In addition, we found that 31 phosphoproteins overlapped between the CUMS-induced and ketamine-induced changes in the mPFC (Supplementary Figure S3A; Supplementary Data Sheet S2). The top enriched GO categories were “cellular component organization” and “cellular component organization or biogenesis” in biological process. “neuron part” and “protein binding” were the top enriched categories in cell component and molecular function, respectively (Supplementary Figure S3B). For the KEGG pathway analysis, we found that “Retrograde endocannabinoid signaling” and “Synaptic vesicle cycle” were the top enriched categories (Supplementary Figure S3C). All other categories from the GO and KEGG analyses are shown in Supplementary Figures S3B,C. In the NAc, 13 phosphoproteins overlapped between the CUMS-induced and ketamine-induced changes (Supplementary Figure S3D; Supplementary Data Sheet S2). After GO analysis, we found that “localization” was the top enriched category in biological process, and “neuron part” and “vesicle” were the top enriched categories in cell components. For molecular function, “lipid binding” was the top enriched category (Supplementary Figure S3E). For the KEGG pathway analysis, the top enriched signaling pathways included the “Wnt signaling pathway” and “Insulin secretion” (Supplementary Figure S3F). All other categories from the GO and KEGG analyses are shown in Supplementary Figures S3E,F.

**Figure 5:**
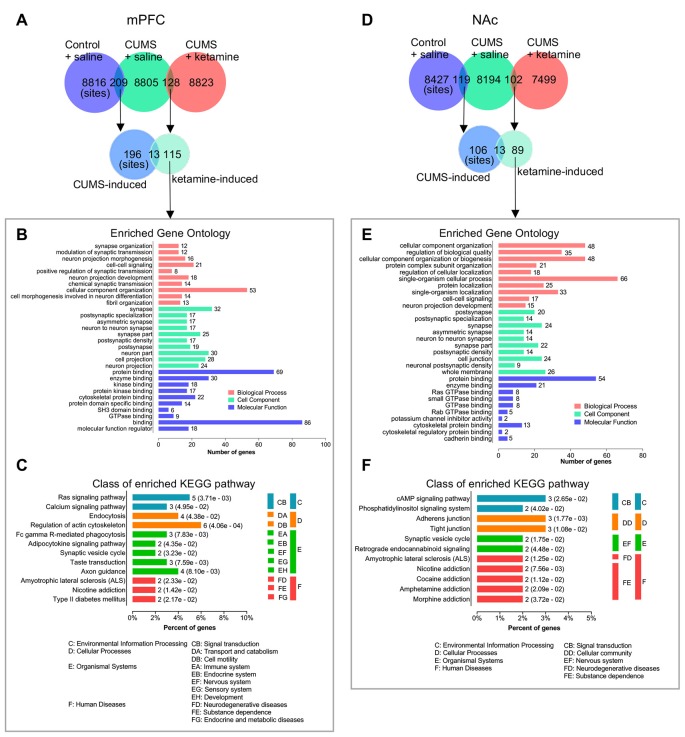
Bioinformatics analysis of the phosphorylation site changes induced selectively by ketamine, but not by CUMS. **(A)** The overlapping of identified phosphorylated sites for indicated experimental groups in the mPFC. **(B,C)** Bioinformatics analysis of the proteins corresponding to phosphorylation sites induced selectively by ketamine, but not by CUMS in the mPFC. The GO and KEGG pathway analyses are shown in **(B,C)** respectively. **(D)** The overlapping of identified phosphorylated sites for indicated experimental groups in the NAc. **(E,F)** Bioinformatics analysis of the proteins corresponding to phosphorylation sites induced selectively by ketamine, but not by CUMS in the NAc. The GO and KEGG pathway analyses are shown in **(E,F)** respectively. Enriched GO analysis shows the 10 most significantly enriched terms of biological process, cell component, and molecular function, respectively. Enriched KEGG pathway analysis indicates the number of involved genes in a specific pathway, and corresponding *p*-values are shown on the right side of the column.

Furthermore, we analyzed the differential phosphorylation changes induced by ketamine, but not by CUMS, as these changes might also be important for the antidepressant effect of ketamine. We identified 115 phosphorylation sites in the mPFC (Figure 5A; Supplementary Data Sheet S3). After GO analysis, we found that several synaptic transmission-related signaling pathways were enriched in biological processes, such as “synapse organization,” “modulation of synaptic transmission,” and “chemical synaptic transmission,” suggesting synaptic changes might be a hallmark of ketamine treatment. For cell component, many synapse-related structure proteins were enriched in “synapse,” “postsynaptic specialization,” “postsynaptic density” and “neuron part,” suggesting that changes in the synaptic structure might be important after ketamine treatment. For molecular function, “binding” was the top enriched category, followed by “protein binding,” “enzyme binding,” and “cytoskeletal protein binding” (Figure 5B), suggesting that a series of intracellular signaling pathways were rapidly activated after ketamine injection (1 h). For KEGG analysis, the top enriched signaling pathways included “Regulation of actin cytoskeleton.” In addition, the “Ras signaling pathway” and “Axon guidance” were enriched (Figure 5C). All other categories from the GO and KEGG analyses are shown in Figures 5B,C. For the NAc, we identified 89 phosphorylation sites that were induced by ketamine, but not by CUMS (Figure 5D; Supplementary Data Sheet S3). After GO analysis, we found that the “single-organism cellular process” was the top enriched category in biological process, followed by “cellular component organization” and “cellular component organization or biogenesis.” For cell component, “whole membrane,” “synapse,” and “cell junction” were among the top categories, followed by “synapse part” and “postsynapse.” For molecular function, we found that “binding” was also the top enriched categories, including “protein binding,” “enzyme binding,” “cytoskeletal protein binding,” and “GTPase binding” (Figure 5E). These results suggested that a series of intracellular signaling pathways were rapidly activated following ketamine injection. For KEGG analysis, we found that the top enriched signaling pathways included “cAMP signaling,” “Adherens junction” and “Tight junction,” followed by “Phosphatidylinositol signaling” and “Synaptic vesicle cycle” (Figure 5F), suggesting that a different set of signaling pathways was activated in the NAc compared to the mPFC following ketamine treatment. All other categories from the GO and KEGG analyses are shown in Figures 5E,F.

In addition, we analyzed the differential phosphoprotein changes induced by ketamine but not by CUMS. We identified 81 phosphoproteins in the mPFC (Supplementary Figure S4A; Supplementary Data Sheet S4). After GO analysis, we found that “positive regulation of biological process,” “cytoplasm,” and “protein binding” were the top enriched categories in biological process, cell component, and molecular function, respectively (Supplementary Figure S4B). We found that “Regulation of actin cytoskeleton” was the top enriched category in KEGG pathway analysis (Supplementary Figure S4C). All other categories from the GO and KEGG analyses are shown in Supplementary Figures S4B,C. In the NAc, we identified 80 phosphoproteins for GO and KEGG pathway analyses (Supplementary Figure S4D; Supplementary Data Sheet S4). For GO analysis, “single-organism cellular process,” “whole membrane,” and “protein binding” were the top enriched categories in biological process, cell component, and molecular function, respectively (Supplementary Figure S4E). We found that the “cAMP signaling pathway,” “Adherens junction,” and “Tight junction” were the top enriched categories in KEGG pathway analysis (Supplementary Figure S4F). All other categories from the GO and KEGG analyses are shown in Supplementary Figures S4E,F.

Nevertheless, we analyzed the overlapping phosphorylation sites between the mPFC and the NAc. We found that only three sites were common for CUMS-induced changes: Dync1li1 (S510), Jag2 (S1125) and Gpm6a (S267; Supplementary Figure S5A). Dync1li1 (S510) and Jag2 (S1125) were up-regulated by CUMS in the mPFC and the NAc, and Gpm6a (S267) was up-regulated in the mPFC but down-regulated in the NAc (Supplementary Figure S5A). Cytoplasmic dynein 1 light intermediate chain 1 (Dync1li1) is a subunit of cytoplasmic dynein that moves cellular components toward the minus ends of microtubules and determines the distribution of vesicular organelles (Tynan et al., 2000; Sivaram et al., 2009). Jagged-2 (Jag2) is one of several ligands that activates Notch and related receptors, and the Notch signaling pathway plays an important role in neuronal plasticity (Shimizu et al., 2000; Monsalve et al., 2014). Neuronal membrane glycoprotein M6-a (Gpm6a) is a transmembrane protein that plays an important role in neurite/filopodium outgrowth and synapse formation (Alfonso et al., 2005). For ketamine-induced changes, there were four sites in common between the two brain regions: Rims1 (S790), Hdgfrp2 (S635), Set (S30) and Arhgap44 (S598; Supplementary Figure S5B). Rims1 (S790) was up-regulated by ketamine in the mPFC and the NAc, Hdgfrp2 (S635) was up-regulated in the mPFC but down-regulated in the NAc, and Set (S30) and Arhgap44 (S598) were down-regulated in both brain regions (Supplementary Figure S5B). Regulating synaptic membrane exocytosis protein 1 (Rims1) is a member of the Ras superfamily and plays an important role in neurotransmitter release (Wang et al., 1997; Calakos et al., 2004). Hepatoma-derived growth factor-related protein 2 (Hdgfrp2) binds to histone marks characteristic of transcriptionally silent chromatin and recruits the homologous recombination repair machinery to active genes silenced upon DNA damage (Baude et al., 2016). Set nuclear proto-oncogene (Set) interacts with numerous proteins involved in histone modification and plays a pivotal role in neurogenesis (Stevens et al., 2018). Rho GTPase activating protein 44 (Arhgap44) is a GTPase-activating protein for the small GTPases RAC1 and CDC42 and is involved in cell spreading and migration (Xu et al., 2017). The few overlapping sites between the two brain regions suggest that they use different signaling mechanisms in response to ketamine treatment.

### Candidate Verification by RNA *in situ* or Western Blot

**Figure 6:**
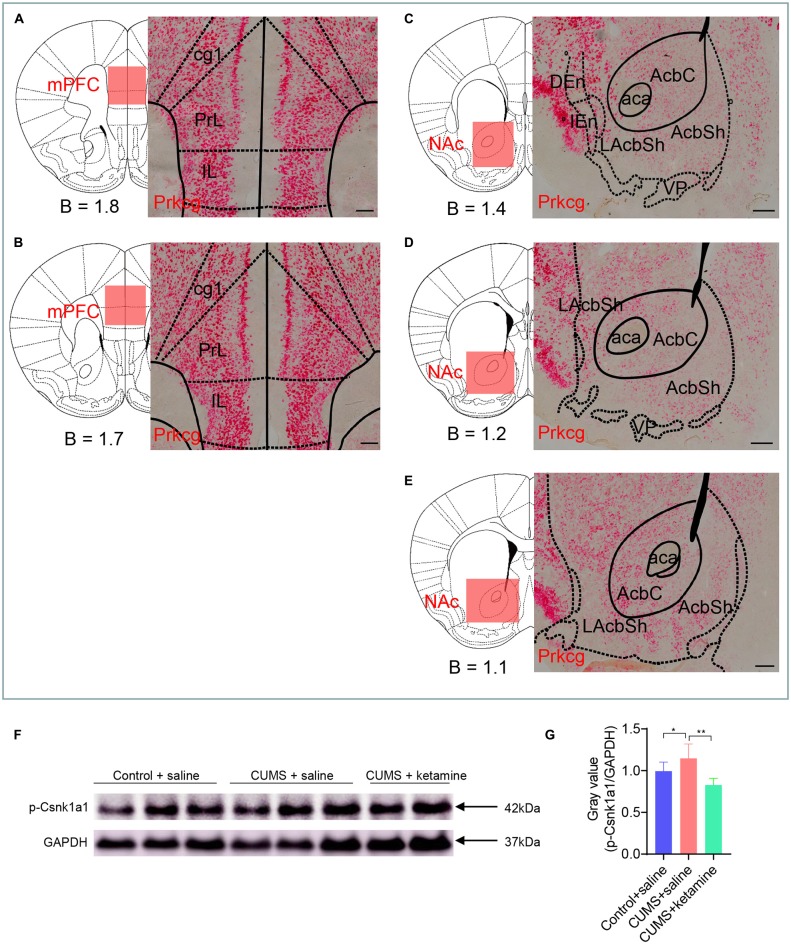
Candidate verification by RNA in situ or western blot. **(A–E)** RNAscope in situ hybridization was performed on coronal sections of wild-type mouse brains in the mPFC and NAc with a Prkcg probe. Prkcg mRNA was expressed in both the mPFC and the NAc. Scale bars are 200 μm. B, bregma; cg1, cingulate cortex, area 1; PrL, prelimbic cortex; IL, infralimbic cortex; aca, anterior commissure, anterior part; AcbC, accumbens nucleus, core; LAcbSh, lateral accumbens shell; AcbSh, accumbens nucleus, shell; DEn, dorsal endopiriform claustrum; IEn, intermediate endopiriform claustrum; VP, ventral pallidum. **(F–G)** Western blot analysis for the phosphorylation of Csnk1a1 at T321. All data are shown as mean ±SEM. *P < 0.05, one-way ANOVA tests for CUMS + saline compared with Control + saline; **P < 0.01, one-way ANOVA tests for CUMS + saline compared with CUMS + ketamine.

We sought to verify the expression and phosphorylation levels of some candidate proteins by RNA *in situ* and western blot. We checked the expression pattern of Prkcg by RNAscope in situ hybridization since it is a kinase that was drastically enriched by CUMS in the mPFC (8.5-fold, Figure 3C) and by ketamine in the NAc (1.47-fold, Figure 3D) and was involved in all four synaptic types (Figures 3A,B). As expected, the RNA *in situ* revealed that Prkcg mRNA was expressed in both the mPFC and the NAc, with a stronger expression in the mPFC (Figures 6A–E). It might be interesting to check the function of this protein kinase in depression in the future.

Besides, we searched antibodies for identified phosphoproteins and found suitable antibodies for p-Csnk1a1 (T321). The level of p-Csnk1a1 (T321) was up- regulated after CUMS treatment and was restored by ketamine (Figures 6F,G), which is consistent with the results from the LC-MS/MS analysis (Supplementary Data Sheet S1). Csnk1a1 is one of the vital components of the Wnt/β-catenin signaling pathway and is a serine/threonine kinase that inhibits β-catenin (Jiang et al., 2018). It is a tumor suppressor gene for colon cancer and melanoma, and controls proliferation through regulating the β-catenin activity (Sinnberg et al., 2010; Elyada et al., 2011). Epithelial cells lacking Csnk1a1 also exhibits many of the characteristics of human colorectal tumors, particularly induces DNA damage responses and cellular senescence (Carreras Puigvert et al., 2013). It might be interesting to check whether this kinase plays a vital role in depression.

## Discussion

Protein phosphorylation, one of the most common intracellular protein modifications, controls a variety of cellular processes, such as kinase-mediated addition of high-energy phosphate groups to serine, threonine, or tyrosine residues on proteins to participate in cells signaling (Martins-de-Souza et al., 2012). Large-scale analyses and quantification of phosphoproteins and their phosphorylation sites using mass spectrometric analysis allow us to systematically analyze the functional role of proteins, such as those involved in signal transduction (Mann et al., 2002). In the present study, we used a highly efficient method to perform phosphorylation proteomic analysis combining TiO_2_ phosphopeptide enrichment with LC-MS/MS analysis. We analyzed 17 samples and identified ~8,800 phosphorylated sites mapping to ~2,900 proteins from the mPFC groups (Figure 1I) and ~8,000 phosphorylated sites mapping to ~2,750 proteins from the NAc groups (Figure 1J). By analyzing these differential phosphorylation sites among Control, CUMS and CUMS + ketamine, we provided a list of phosphoproteins and their phosphorylation sites that will help elucidate the signaling changes in different brain regions in depressive conditions and following ketamine treatment. Noticeably, it would be interesting to include a ketamine-treated control to see whether ketamine treatment-induced changes are specific to depression states over normal states. Also, it would be more informative to include a regular protein mass spectra for comparison to check whether changes in protein concentration contribute to the changes in protein phosphorylation.

Intriguingly, our findings support the role of synaptic function in depression induction and treatment, as shown by PPI networks. Unexpectedly, we found that “synapse” was the common feature, including glutamatergic, GABAergic, cholinergic, dopaminergic, and synaptic vesicle cycle, as indicated (Figures 2, 3). Another interesting finding is the opposite changes in phosphorylation between the CUMS-induced and ketamine-induced groups, suggesting that the altered phosphorylation induced by CUMS was reversed by ketamine treatment. Consistent with these changes, several corresponding proteins have been reported to play a role in depression. For example, Cacna1a encodes the transmembrane pore-forming subunit of the P/Q-type or CaV2.1 voltage-gated calcium channel, which regulates the efficiency of synaptic transmission at presynaptic membrane active zones (Lübbert et al., 2017, 2019). The protein levels of Map2 in the hippocampus of CUMS model rats were down-regulated, which could be reversed by Ro41-5253 or fluoxetine treatment (Ke et al., 2019). Another interesting protein is Prkcg, a PKC that is activated by Ca^2+^ and diacylglycerol in the presence of phosphatidylserine (Saito and Shirai, 2002). It is expressed in both the mPFC and the NAc (Figures 6A–E). Selective blockade of the PLCb1-PKCg signaling pathway produced an antidepressant-like phenotype in mice (Galeotti and Ghelardini, 2011), consistent with the significant increase of Prkcg (S322) induced by CUMS in the mPFC in our data (Figure 3C). However, Prkcg (S342) showed the opposite change in the NAc (Figure 3D), which implies that different phosphorylation sites in different brain regions may play different roles in depression. Furthermore, Prrt2 (S244) in the NAc attracted our attention due to its dramatic changes, although this protein’s function is still unclear. Some evidence suggests a functional role at synapses, such as its coexpression with synaptic markers (Lee et al., 2012) or interaction with synaptic vesicle cycling (Rossi et al., 2016). Interestingly, our study also found that prrt2 is involved in the synapse-related process (Figure 4G), such as neurotransmitter transport and synaptic vesicle exocytosis.

Notably, regarding the differential phosphorylation changes induced by ketamine, but not by CUMS, “synapse” still accounts for a large proportion of the categories from the GO analysis (Figures 5B,E). Consistent with our study, many previous studies have shown that ketamine can reverse the synaptic deficits caused by stress (Li et al., 2011; Belujon and Grace, 2014; Ghosal et al., 2017). Surprisingly, only a few phosphorylation sites were overlapping between the mPFC and the NAc induced by CUMS and ketamine, respectively (Supplementary Figure S5). We found that only three sites, Dync1li1 (S510), Jag2 (S1125) and Gpm6a (S267), were in common between the two brain regions induced by CUMS (Supplementary Figure S5A). For ketamine-induced changes, there were four sites in common: Rims1 (S790), Hdgfrp2 (S635), Set (S30) and Arhgap44 (S598; Supplementary Figure S5B). These findings may indicate that the two brain regions respond differently to depression and ketamine treatment.

Here, we identified the global phosphoproteomic changes in CUMS-induced depressive states and ketamine treatment. Through different analyses, we found that many of the phosphorylated proteins quantified in our study were involved in synaptic functions, including glutamatergic, GABAergic, cholinergic and dopaminergic synapses. The CUMS-induced phosphorylation changes were reversed by ketamine treatment. Hence, our research further supports the role of the synaptic machinery in neuropsychiatric disorders and predicts signaling mechanisms for treating depression *via* ketamine, which may provide a foundation for the development of novel treatment strategies (Duman and Aghajanian, 2012; Martins-de-Souza et al., 2012; Duman et al., 2016).

## Data Availability Statement

The mass spectrometry proteomics data have been deposited to the ProteomeXchange Consortium (http://proteomecentral.proteomexchange.org) *via* the iProX partner repository (Ma et al., 2019) and the dataset identifier is PXD016302.

## Ethics Statement

The animal study protocol was reviewed and approved by the Animal Care and Use Committee of ShanghaiTech University, Shanghai Model Organisms Center, Inc.

## Author Contributions

YX and HL performed most of the experiments. HL and YZ performed the behavioral evaluations. YX, HL, ZS, WY, and XN prepared the phosphoproteomics samples and LC-MS/MS analysis. YX, JZ, ZL, XF, YS, and HT performed the bioinformatics analysis. WLS, JH, JY, YX, HL, and WS designed the experiments. YX, HL, JH, and WLS wrote the manuscript.

## Conflict of Interest

JZ was employed by the company Delta Omics Inc. The remaining authors declare that the research was conducted in the absence of any commercial or financial relationships that could be construed as a potential conflict of interest.

## Footnotes

^1^http://www.omicsbean.com:88/

^2^https://www.genome.jp/kegg/pathway.html
