# Supplementary material for: A Brain Signaling Framework for Stress-Induced Depression and Ketamine Treatment Elucidated by Phosphoproteomics
Source: Front Cell Neurosci. 2020 Apr 7;14:48. doi: 10.3389/fncel.2020.00048 (PMC7156020; doi:10.3389/fncel.2020.00048)
Supplement: Supplementary file 5 [file Image_1.pdf]

Figure S1 Pearson correlation analysis between experiments

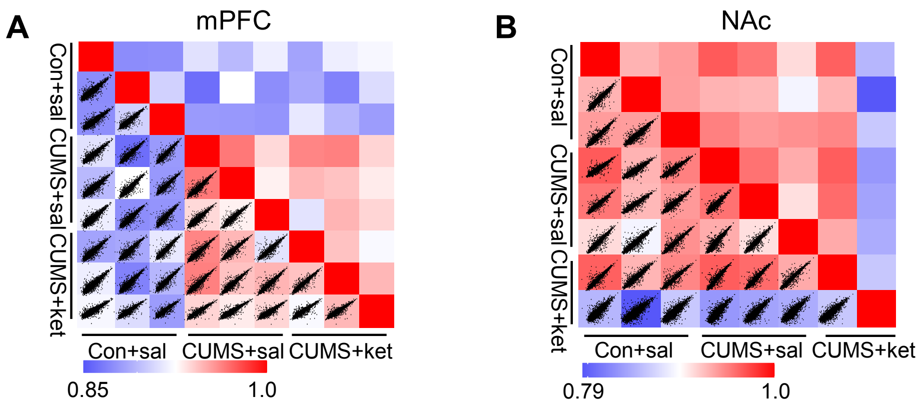

FIGURE S1 Pearson correlation analysis between experiments. (A-B) Pearson correlation analysis between each biological replicate of the mPFC **(A)** and the NAc **(B)** indicates a high correlation between experimental repeats. Scatter plots of relative intensities of each phosphorylated site in Control + saline, CUMS + saline and CUMS + ketamine are shown. The heatmap of Pearson correlation values are mirrored diagonally, and the biological replicates of each condition are colored according to the correlation coefficient values. The scales are indicated below the heatmap.

Figure S2 The number of quantified differential phosphorylated sites from CUMS + ketamine vs. Control + saline

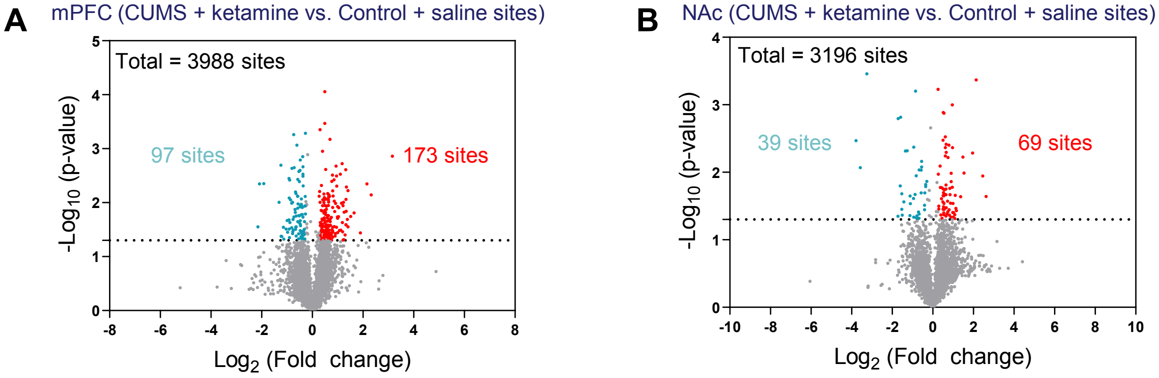

FIGURE S2 The number of quantified differential phosphorylated sites from CUMS + ketamine versus Control + saline. (A-B) The number of quantified differential phosphorylated sites from CUMS + ketamine vs. Control + saline in the mPFC **(A)** and the NAc **(B)**. The ratio of 1.2 fold change and the probability of 0.05 as a cut-off for differential expressed sites with statistical significance. The differential phosphorylated sites are shown in red (up-regulated) and blue (down-regulated).

Figure S3 Bioinformatics analysis of the common change of phosphoproteins between CUMS-induced and ketamine-induced

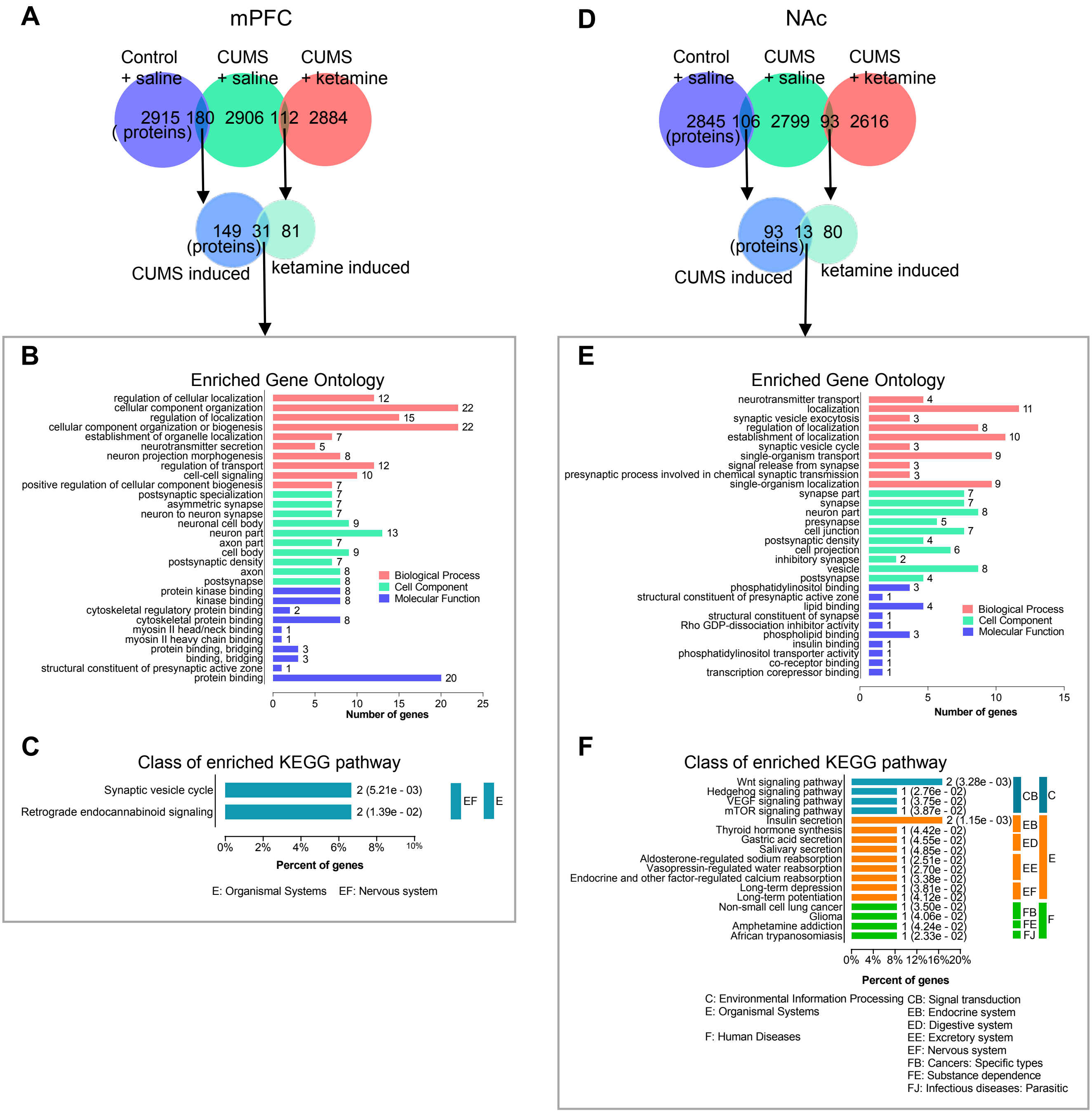

FIGURE S3 Bioinformatics analysis of the common change of phosphoproteins between CUMS-induced and ketamine-induced. (A) The overlapping of identified phosphoproteins for indicated experimental groups in the mPFC. **(B-C)** Bioinformatics analysis of the common phosphoproteins between CUMS-induced and ketamine-induced in the mPFC. The GO and KEGG pathway analyses are shown in **(B)** and **(C)** respectively. **(D)** The overlapping of identified phosphoproteins for indicated experimental groups in the NAc. **(E-F)** Bioinformatics analysis of the common phosphoproteins between CUMS-induced and ketamine-induced in the NAc. The GO and KEGG pathway analyses are shown in **(E)** and **(F)** respectively. Enriched GO analysis shows the ten most significantly enriched terms of biological process, cell component, and molecular function, respectively. Enriched KEGG pathway analysis shows the number of involved genes in a specific pathway, and corresponding *p*-values are shown on the right side of column.

Figure S4 Bioinformatics analysis of the phosphoproteins induced selectively by ketamine, but not by CUMS

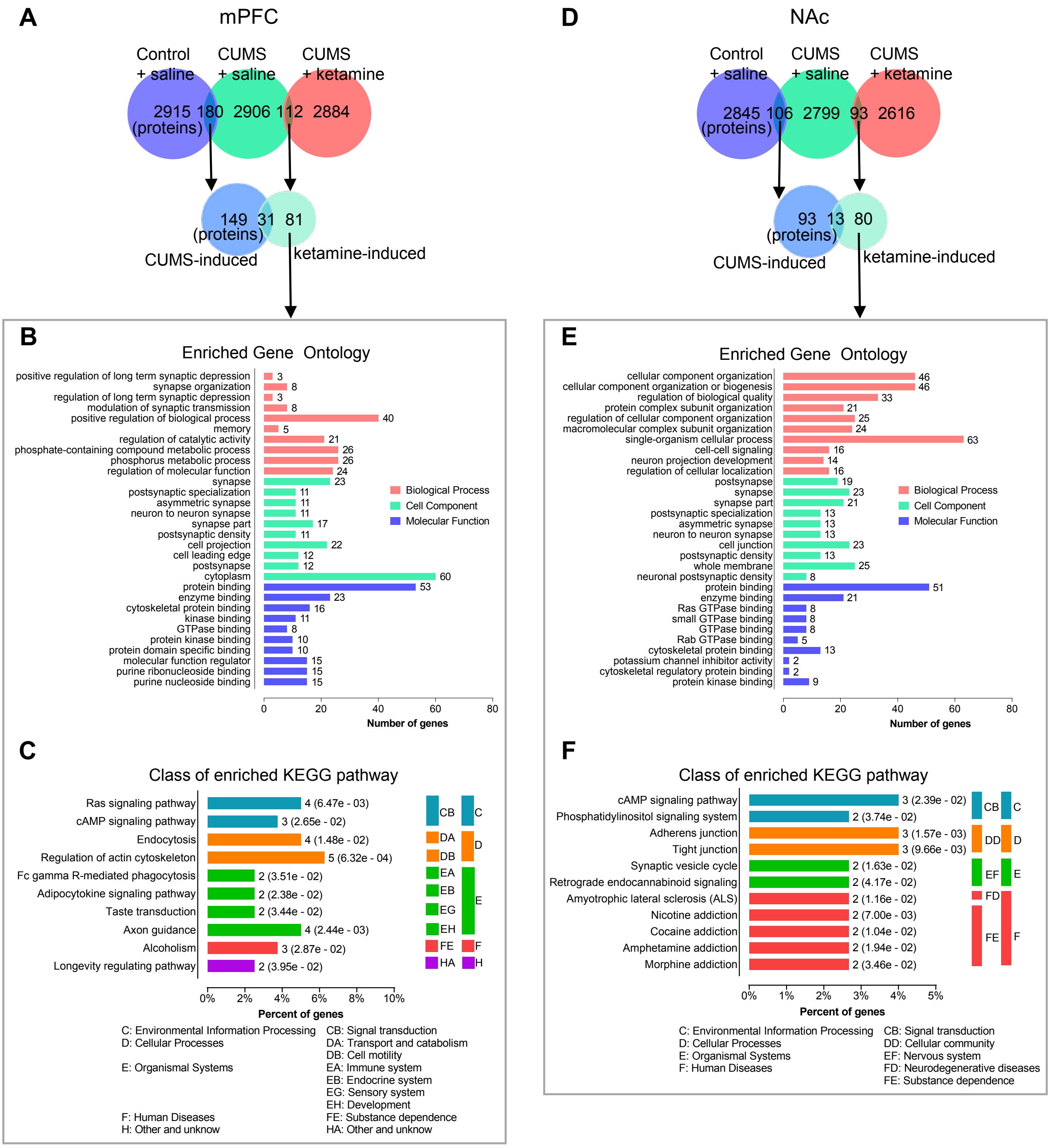

FIGURE S4 Bioinformatics analysis of the phosphoproteins induced selectively by ketamine, but not by CUMS. (A) The overlapping of identified phosphoproteins for indicated experimental groups in the mPFC. **(B-C)** Bioinformatics analysis of the phosphoproteins induced selectively by ketamine, but not by CUMS in the mPFC. The GO and KEGG pathway analyses are shown in **(B)** and **(C)** respectively. **(D)** The overlapping of identified phosphoproteins for indicated experimental groups in the NAc. **(E-F)** Bioinformatics analysis of the phosphoproteins induced selectively by ketamine, but not by CUMS in the NAc. The GO and KEGG pathway analyses are shown in **(E)** and **(F)** respectively. Enriched GO analysis shows the ten most significantly enriched terms of biological process, cell component, and molecular function, respectively. Enriched KEGG pathway analysis shows the number of involved genes in a specific pathway, and corresponding *p*-values are shown on the right side of column.

Figure S5 The common phosphorylation sites between the mPFC and the NAc induced by CUMS or ketamine

A CUMS-induced

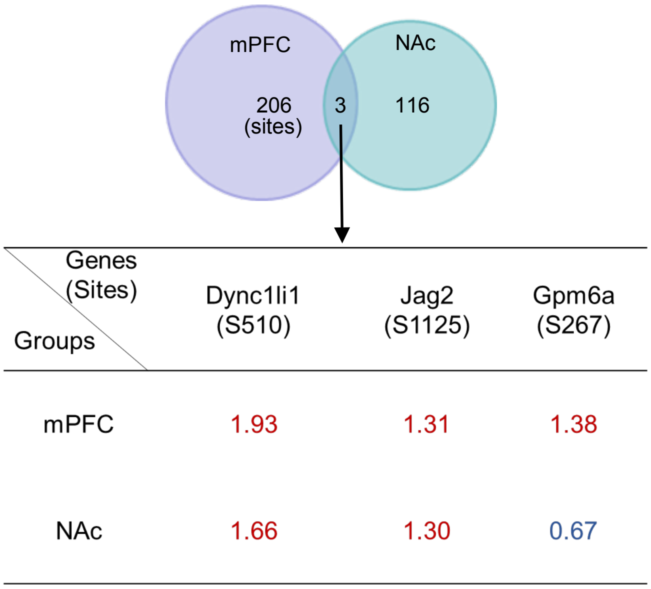

Genes (Sites) / Groups	Dync1li1 (S510)	Jag2 (S1125)	Gpm6a (S267)
mPFC	1.93	1.31	1.38
NAc	1.66	1.30	0.67

B ketamine-induced

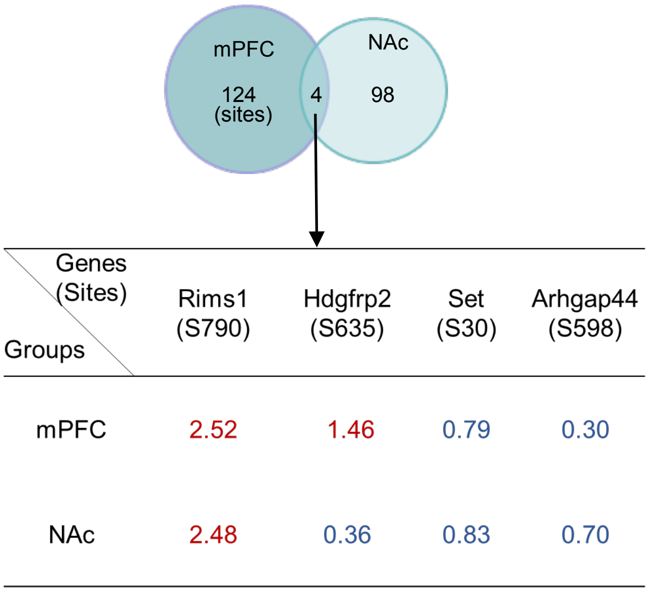

Genes (Sites) / Groups	Rims1 (S790)	Hdgfrp2 (S635)	Set (S30)	Arhgap44 (S598)
mPFC	2.52	1.46	0.79	0.30
NAc	2.48	0.36	0.83	0.70

Red color : up-regulated   Blue color : down-regulated

FIGURE S5 The common phosphorylation sites between the mPFC and the NAc induced by CUMS or ketamine. (A) The overlapping of identified phosphorylated sites induced by CUMS between the mPFC and the NAc. The genes corresponding to the common phosphorylation sites are listed in the table. The red and blue colors indicate up and down-regulated, respectively. **(B)** The overlapping of identified phosphorylated sites induced by ketamine between the mPFC and the NAc. The genes corresponding to the common phosphorylation sites are listed in the table. The red and blue colors indicate up and down-regulated, respectively.

Table S1 The overlapped phosphorylation sites between each replicate

Brain regions	Groups	Replicate	Identified Phosphorylation Sites	Overlapped Phosphorylation Sites	Overlapped /Identified (%)
PFC	Control + saline	1	8417	#1&#2= 6852	79
		2	8995	#1&#3= 6783	78
		3	9035	#2&#3= 7233	80
	CUMS + saline	1	9074	#1&#2= 7188	81
		2	8772	#1&#3= 7084	80
		3	8570	#2&#3= 6971	80
	CUMS + ketamine	1	8705	#1&#2= 7089	81
		2	8715	#1&#3= 7188	81
		3	9048	#2&#3= 7240	82
NAc	Control + saline	1	8567	#1&#2= 6899	81
		2	8507	#1&#3= 6632	79
		3	8207	#2&#3= 6526	78
	CUMS + saline	1	8196	#1&#2= 6675	80
		2	8418	#1&#3= 6300	78
		3	7969	#2&#3= 6441	79
	CUMS + ketamine	1	8248	#1&#2= 5099	68
		2	6749		

Note. We showed the number of identified phosphorylation sites in each repeat group, the number of overlapped phosphorylation sites between each two repeat groups, and the percentage of the number of overlapped phosphorylation sites to mean identified phosphorylation sites of the two repeats.

Table S2 The phosphorylated genes/sites that ketamine reversed to normal in the mPFC

Groups \ Genes (Sites)	Cacna1a (T1888)	Rims1 (S790)	Srr (S71)	Ttbk1 (S484)	Ubr5 (S1549)	Epb4.1l4b (S114)	Iqsec1 (S401)	Lmnb2 (S427)	Srrm1 (S429)	Ube3a (S8)
CUMS + saline vs. Control + saline	0.71	0.47	0.63	0.74	0.67	1.44	1.35	1.45	1.34	1.38
CUMS + ketamine vs. CUMS + saline	1.41	2.52	1.51	1.31	1.51	0.79	0.83	0.75	0.81	0.75

Note. The following two criteria were used for 'reversed' phosphorylation sites: 1. *P* value $<$ 0.05; 2. The multiplied fold changes of CUMS + saline vs. Control + saline and CUMS + ketamine vs. CUMS + saline was in between 0.83 with 1.2. The red and blue colors indicate up- and down-regulated.

Table S3 The phosphorylated genes/sites that ketamine reversed to normal in the NAc

Groups \ Genes (Sites)	Crocc (S501)	Crocc (T494)	Prkcg (S342)	Arhgap32 (S856)	Cadps (S89)	C2cd2l (S468)	Csnk1a1 (T349)	Rims2 (S1137)
CUMS + saline vs. Control + saline	0.73	0.73	0.79	1.22	1.42	1.36	1.88	1.25
CUMS + ketamine vs. CUMS + saline	1.52	1.52	1.47	0.79	0.79	0.75	0.59	0.75

Note. The following two criteria were used for 'reversed' phosphorylation sites: 1. *P* value $<$ 0.05; 2. The multiplied fold changes of CUMS + saline vs. Control + saline and CUMS + ketamine vs. CUMS + saline was inbetween 0.83 with 1.2. The red and blue colors indicate up- and down-regulated.
